# Heterotopic Ossification in Orthopaedic and Trauma surgery: A Histopathological Ossification Score

**DOI:** 10.1038/s41598-019-54986-2

**Published:** 2019-12-05

**Authors:** M. Ohlmeier, V. Krenn, D. M. Thiesen, N. A. Sandiford, T. Gehrke, M. Citak

**Affiliations:** 1 Department of Joint Surgery, Helios ENDO-Klinik Hamburg, Holstenstrasse 2, 22767 Hamburg, Germany; 2 MVZ-Zentrum für Histologie, Zytologie und Molekulare Diagnostik, Trier, GmbH, Max-Planck-Straße 5, 54296 Trier, Germany; 3 Joint Reconstruction Unit, Southland Hospital, Kew Rd, Invercargill, 9812 New Zealand

**Keywords:** Trauma, Experimental models of disease, Preclinical research

## Abstract

Heterotopic Ossification (HO) is a potential long-term complication in orthopaedic surgery. It is commonly classified according to the Brooker classification, which is based on radiological findings. To our knowledge the correlation of histological features to the Brooker grade is unknown as is the association between HO and the indication for revision. The aim of this paper is to analyze the ossification grade of HO tissue in patients undergoing revision hip and knee arthroplasty and to propose a histologically based classification system for HO. We also assess the relationship between the grade of HO and the indication for revision (septic and aseptic revision). From January to May 2019 we collected 50 human HO samples from hip and knee revision arthroplasty cases. These tissue samples were double-blinded and sent for histopathological diagnostic. Based on these results, we developed a classification system for the progression of HO. The grade of ossification was based on three characteristics: Grade of heterotopic ossification (Grade 1–3), presence of necrosis (N0 or N1) and the presence of osteomyelitis (HOES-Score Type 1 to 5). Demographic data as well as surgical details and indication for surgery was prospectively collected from clinical records. Fifty tissue samples were harvested from 44 hips and 6 knee joints. Of these 33 exhibited Grade I ossifications (66%), followed by 11 Grade II (22%) and one Grade III (2%). Necrosis was noted in two tissue samples (4%) and 2 more had osteomyelitis findings according to HOES-Score. Six samples (12%) with radiologically suggestive of HO turned out to be wear-induced synovitis, SLIM Type 1. Of these cases 16 were septic (32%) and 34 aseptic (68%) revisions. Most of the HO tissue samples were classified as a low-grade. High-grade ossification-Score is rare. Higher grades of ossification seem to be associated with septic revision cases. Wear-induced synovitis potentially influences HO development. A histological scoring system for ossification grading can be derived from the data presented in this study.

## Introduction

**Figure 1:**
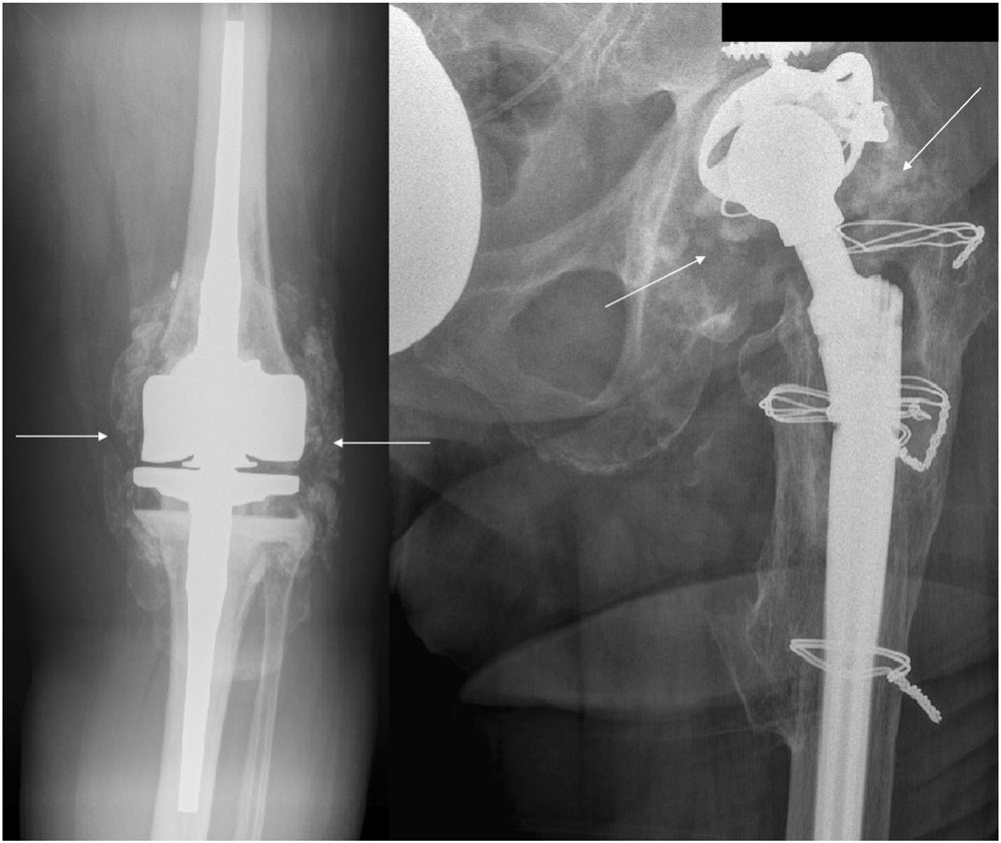
Heterotopic Ossification in revision knee and hip arthroplasty preoperatively.

Heterotopic Ossification (HO) or Myositis ossificans (MO) is a recognized complication of total hip and total knee arthroplasty surgery. It is more commonly noted in revision arthroplasty cases and its etiology is poorly understood and thought to be multifactorial^1–3^ (Fig. 1). The incidence of HO has been reported to be as high as 26–41%^4–6^ and can lead to painful restriction of joint motion^7,8^. Effective therapy strategies have been found with the use of nonsteroidal antiinflammatory drugs (NSAID) and focused radiotherapy^9–11^.

It has been proposed that mesenchymal progenitor cells are responsible for the development of HO^2,12^. These progenitor cells transform into osteoblasts under the influence of morphogenes^2^. The concentrations of morphogene can be elevated in muscle and soft tissue after any kind of trauma, so that an enchondromal ossification leads to formation and maturation of lamellar bone^2^. This is the proposed mechanism by which HO occurs following major procedures such as revision total joint arthroplasty, particularly cases with significant surgical dissection and surgical trauma such as septic cases^13^.

Several classification systems for HO have been proposed^14–16^. The Brooker classification is the most popular of these and has been used since 1973. It is based on observations from a cohort of 100 patients^17^. In this study, patients were evaluated 6 months postoperatively after THA. The Harris Hip Score and radiographic incidence of HO were noted. HO was found in 21% of the patients. Since this original description, HO has been described in different joints and muscle groups. The Brooker classification was not described for joints apart from the hip and existing classifications do not account for HO in other regions^18–24^.

The aim of this study is to propose a histopathological Ossification Score that allows to express ossification grades in any human joint. Furthermore, these characters involve not only the ossification grade but also necrosis and osteomyelitis findings as well as the SLIM-classification (LIT) and the particle algorithm (LIT) in case of peri-implant tissue and the identification of particles deposits^25^.

## Patients and Methods

### Patients

This study was approved by the Institutional Review Board of Hamburg University College of Medicine (Weidestraße 122 b, 22083 Hamburg, Germany; Date of approval: 07 January 2019). All study procedures followed the tenets of the Declaration of Helsinki and written informed consent was obtained from all participants, no patients under an age of 18 years were included. The study used an experimental design, experimental methods were carried out in accordance with the ethical guidelines and regulations of the medical council Hamburg.

### Data collection

This study was performed between January and May 2019. Fifty human tissue samples were harvested from 44 hip (88%) and 6 knee (12%) joints. All tissue samples were taken from patients undergoing revision hip and knee arthroplasty. These patients underwent revision surgery either due to periprosthetic joint infection, implant loosening (septic or aseptic) or excision periprosthetic heterotopic bone. All tissue samples were double-blinded and sent for histopathological processing.

We also prospectively collected the following patients’ data from available medical records as well as our electronic database: age, sex, body mass index (BMI), length of hospital stay, postoperative intensive care unit (ICU) stay, preoperative symptoms of systemic inflammatory reaction including night sweats, fever, shivers, and/or unintended weight loss, preoperative C-reactive protein (CRP) and white blood cell count (WBC). Patients’ characteristics are presented in Table 1. Joint-related data including the number of prior joint surgeries and the presence of sinus tracts were also documented. In-hospital complications such as prolonged wound drainage, massive hemarthrosis necessitating aspiration, hip dislocation, other medical joint-nonrelated complications, the need for allogeneic blood transfusion and surgery-related mortality were carefully noted.

**Table 1:** Patients’ characteristics presented in dependence of Ossification.

Descriptive measures of continuous variables in dependency of Heterotopic Ossifications										
variable	HO	n	mean	SD	min	25% perc.	median	75% perc.	max	p*
Age [yrs]	yes	45	70.4	10.7	40	66	69	78	90	0.528
	no	5	72.6	17.2	52	56	82	85	88	
BMI [kg/m^2^]	yes	35	28.76	5.27	19.4	24.8	27.7	32.7	38.9	0.683
	no	5	27.56	4.21	24.3	25.2	25.8	27.8	34.7	
Years since last operation of same joint	yes	44	9.7	8.3	0	2	8	17	28	0.443
	no	4	13.5	11.1	3	5	12	23	27	
CRP [mg/l]	yes	45	14.16	22.30	0.3	1.6	4.2	15.7	91.1	0.185
	no	5	15.95	14.54	2.4	8.2	11.0	18.2	39.9	
Leucocytes [x10/S/9]	yes	45	7.66	2.42	3.7	6.0	7.6	8.8	14.5	0.155
	no	5	6.25	2.81	3.7	4.6	5.9	6.1	11.0	
Hemoglobin [g/dl]	yes	45	12.59	2.42	7.6	10.8	13.0	14.6	15.9	0.106
	no	5	10.90	1.72	8.1	10.9	11.2	11.5	12.8	

HO = heterotopic ossification.

*p value of Mann-Whitney-U test.

#### Histopathological processing of the tissue samples and staining techniques

Histopathological processing of the tissue samples: The macroscopic, gross examination, tissue sampling, the processing of the samples including tissue sample paraffin-processing, decalcifying procedures, histochemical methods diagnostic histopathological classification had been performed in automatic and semi-automatic systems under a certified and accredited framework (quality standard according to DIN: DIN EN ISO/IEC 17020:2012, registry number: D-IS-21311-01-00).

Decalcification: The decalcification was carried out by means of acid (7,5% hydrochloric acid). The ratio of decalcification liquid volume to tissue sample volume was about 1:20. The reaction temperature was room temperature with an incubation time of about 1 to 3 days. The consistency of the samples had been checked every 12–24 hours carrying out a very small, superficial cut at the periphery of the samples using a scalpel in order to improve the applicability for further cutting procedure in the microtome.

#### Gross examination of tissue samples

Soft tissue fraction and osseous tissue fraction: After fixing in buffered formalin (4%) for at least 24 hours, tissue samples were cut representatively according to previously described recommendations which guaranties a complete and correct histopathologic diagnosis^25^. Only in cases of large tissue samples (more than 10 mm in diameter) osseous tissue had been separated in principle from adhering soft tissue and embedded separately, so that a soft tissue fraction (1.1) and an osseous tissue fraction (1.2) were generated which guaranties a complete histopathologic evaluation of osseous tissue as well as soft tissue. Tissues samples with a size up to 20 × 20 × 10 mm where completely embedded, large tissue samples where representatively embedded, including at least 50% of the tissue mass^26^. All these methods follow the published recommendations which are part of the DGORh, DGOOC and IAP (German section) recommendations for bone tissue processing in non-neoplastic orthopedic pathology^26^.

Histopathology and tissue staining: The microtomised tissue sections with a section thickness of 1–3 µm were stained with haematoxylin and eosin (H&E) in a fully automated system (Leica- PELORIS® or SACURA-VIP-6 -AI®) with a barcode-tracking system, Roche, VANTAGE workflow solution®). These systems are closed and pressure-vacuum based systems with very low emissions ensuring additionally high quality and high consistency as well reproducibility of the staining results. In some cases, a periodic-acid-Schiff (PAS) as well Preussian-blue-reaction staining was additionally performed. These techniques were carried out after evaluating the HE stained slides especially in cases with intense inflammatory infiltration and intracellular, granular deposits allowing the discrimination and specification of neutrophilic segmented granulocytes and intracellular haemosiderin deposits, and by this way ruling out intracytoplasmic particles from prosthetic materials (for example: metal particles or zirconium dioxide particles).

#### Structure of the ossifications-score (OS)

General considerations: The ossification-score (OS) is a semiquantitative score evaluating three different pathogenetic tissue characteristis. These are heterotopic ossification, necrosis and osteomyelitis. The first two characteristics (ossification and necrosis) comprise the newly defined score whereas the last quality (osteomyelitis) is scored according to the HOES (Histopathological Osteomyelitis Evaluation Score) which had been already accepted in routine diagnostic histopathology for typing of infectious osteomyelitis^27^. The HOES allows a histopathological stratification in following 5 types: I- Signs of an acute osteomyelitis, II- Signs of a chronically florid (that is to say active) osteomyelitis, III- Signs of a chronic osteomyelitis, IV- Signs of a subsided (calmed) osteomyelitis and V- No signs of osteomyelitis. If peri-implant synovial tissue (Synovial Like Interface Membrane, SLIM) was included in the tissue sample typing of peri-implant tissue and the identification of particles deposits was evaluated according to the SLIM-consensus classification (LIT) which defines 7 different types of the periimplant synovial membrane (Synovial Interface Membrane): SLIM Typ 1 particle induced, Type 2 infection induced, the combination of particle induced and infection, SLIM Typ 3, SLIM Typ 4, neither particle induced nor infection, SLIM Type 5, endoprosthetic-associated arthrofibrosis, Type 6 particle-induced immunological, inflammatory and toxic mechanisms (adverse reactions). In cases of particulate deposits, the particle algorithm had been used^28^ which allows a descriptive particle identification of endogenous and exogenous particulate materials and is the basis for particle diagnosis in peri-implant tissue. Both published scoring and typing systems are well accepted scoring systems in diagnostic histopathology^25,29,30^.

Scoring principles: The proposed scoring principles of the Ossification-Score are the evaluation of heterotopic ossification and the evaluation of necrosis. Ossification is evaluated on a three-step evaluation, whereas necrosis on a two-step modality. Heterotopic ossification is defined by the detection of osseous tissue (non-osteon and osteon bone) embedded in the peri-implant fibrous tissue compartment. Semiquantitative and graduated scoring (i.e. three step evaluation) is a general principle of diagnostic histopathology^25,29^. For the detection of score features in conventionally stained HE-slides the objective-magnification of 20x was used (area size about: 1.3 mm^2^). Polarization analysis is necessary for bone tissue typing (lamellar and non-lamellar bone) and for particle definition. The Quantitative definitions concerning the bone formation and numbers of cells per HPF had been defined after subsequent evaluation of all tissue samples and defining retrospectively a quantitative three-step modality. PAS-staining as well as Prussian-blue-reaction may be used for analysis of inflammatory infiltrate and particle-deposit identification.

#### Ossification-score; grades of heterotopic ossification 1 to grade 3 (low, moderate and high)

**Grade 1**: Low bone formation, low bone destruction, low inflammatory infiltration.

**Grade 2**: Moderate bone formation, moderate bone destruction, moderate inflammatory infiltration.

**Grade 3**: High bone formation, high bone destruction, high inflammatory infiltration

#### Quantitative definitions

**Bone formation** (heterotopic ossification):

**Low** (=1): Area of bone formation is lower than one third of the evaluated area.

**Moderate** (=2): Area of bone formation is between one and two third of the evaluated are

**High** (=3): Area of bone formation is more than two third of the evaluated area.

#### Bone destruction

**Low** (=1): low quantity of osteoclast and osteoblast (fewer than 5/HPF)

**Moderate** (=2): moderate quantity of osteoclast and osteoblast (5 to 10/HPF)

**High** (=3): high quantity of osteoclast and osteoblast (more than 10/HPF)

#### Inflammatory infiltration

**Low** (=1): low Inflammatory infiltration (fewer than 5 leucocytes/HPF)

**Moderate** (=2): moderate inflammatory infiltration (5 to 10 leucocytes/HPF)

**High** (=3): high inflammatory infiltration (more 10 leucocytes/HPF)

Non-existent=0

This finding is generally possible but had not been included in this study since all tissue samples had been exclusively taken from regions with pathologic tissue alterations.

#### Qualitative definitions

**Ossification**: Heterotopic ossification is defined by non-lamellar fibrous bone tissue embedded in fibrous tissue characterized morphologically by irregular distributed osteocytes, irregular fibrils which are visualized by polarization optic analysis.

**Destruction**: Destruction is defined by on a cellular level the detection of osteoclasts, whereas osteoblasts are more the cellular substrate of bone turnover which however accompanies osteoclastic cells.

**Inflammatory infiltration**: Inflammatory infiltration is defined by the inflammatory infiltration of leucocytic including the whole spectrum of infiltrating leucocytes ranging from polymorphic segmented neutrophilic granulocytes to mononuclear lymphocytes, macrophages and histiocytic cells.

#### Necrosis

**Qualitative definition**: Necrosis is defined under HE-conditions as eosinophilic areas showing no vital cells respectively cells in state of apoptosis.

#### Quantitative definition

N-0: No Necrosis: No necrotic tissue is detected, very focal areas below 5% are scored as no necrosis.

N-1: Necrosis: Necrotic tissue is detected in more than 5% of the tissue area. 5% had been defined as the cut off since a very low content of necrosis in the sense of very small areas of necrosis may be found in nearly every form of inflammatory infiltration.

#### Osteomyelitis

**Figure 2:**
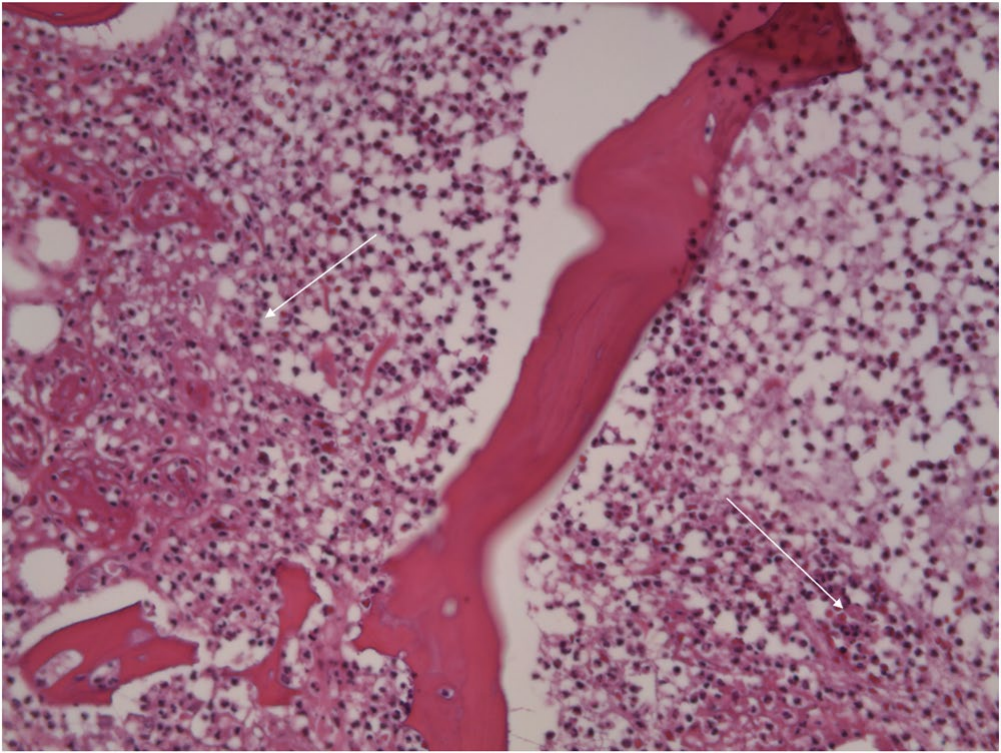
HOES I (Signs of acute Osteomyelitis) with dense accumulations of intramedullary segmented neutrophilic granulocytes. H&E staining, original magnification approximately 200x.

Evaluation of Osteomyelitis had been carried out according to the HOES^27^. The features of the HOES are described in the text. According to this definition, the HOES is an independent evaluation to the above described ossification score (OS) which should give further information in respect to a bacterial infection of the bone tissue. Figure 2 demonstrates a HOES 1 that is “Signs of acute Osteomyelitis” characterized by dense accumulation of intramedullary located segmented neutrophilic granulocytes.

#### Recommendation for the reporting of the ossifications-score (OS)

This score is reported in a simple formula: **Ossification-Score** (Grade 1 to Grade 3), **Necrosis** (N-0: No Necrosis, N-1: Necrosis) and **HOES** (I to V). If peri-implant tissue or synovial tissue is presented the **SLIM types** are listed (type I to type VI) and if **particles** are detectable the particle-identities are described according to **the particle algorithm**.

### Statistical analysis

The distributions of the continuous variables are presented by count (N), mean, standard deviation (SD), extrema (min, max), quartiles (25th percentile, 75th percentile) and median (median). The distribution of categorical data is described by absolute and relative frequencies.

The distributions of a continuous variable of two independent groups were compared using the Mann–Whitney-U test due to small groups. For the comparison of categorical variables Fisher’s exact test was performed. All tests were two-tailed. The analysis has an explorative character. The p values are interpreted in a descriptive manner accordingly. All data were processed using statistical analysis software (Statistica, Version 13.2, Tulsa, Oklahoma, USA).

## Results

Most of the heterotopic ossifications were Grade I results (33 samples, 66%), followed by 11 Grade II (22%) and 1 Grade III (2%) results. Furthermore, we had 2 tissue samples with necrosis (4%) and 2 showing osteomyelitis results according to HOES-Score, one Type II (2%) and one Type III (2%). Under the 50 tissue samples there were 6 samples with preoperative radiological suspect of HO which turned out to be wear-induced synovitis, SLIM Type 1 (12%). Looking at the Brooker Score, we had six Grade I, fifteen Grade II, five Grade III and two Grade IV patients. Another five samples were taken out of knee joints, which are not scorable in the currently used Brooker score. Detailed information can be found in Table 2. All tissue samples were harvested from total hip and knee revision arthroplasty cases. Sixteen (32%) of them were septic revision cases and 34 (68%) were aseptic surgeries. Septic cases were defined as patients undergoing revision surgery with preoperative germ proof taken via aspiration. Seven out of these 16 patients (43.8%) showed bacterial colonization intraoperatively. Thirty-three patients (66%) received nonsteroidal antiinflammatory drugs (NSAID) postoperatively. Looking at potential risk factors for a certain Grade of ossification, we did not find any significant influencing factors, especially concerning CRP levels, gender, time interval since last surgery on the affected joint, intraoperative Microbiology etc.. Further results are given in Tables 1 & 3. Special attention was made on the use on NSAIDs and a potential impact on the three presented variables (Grade of Ossification, Necrosis and Osteomyelitis). There was no significant correlation, though all our necrosis findings were associated with missing NSAID prescription and the majority of Grade I ossifications received NSAID (Table 4

**Table 2:** Listing of Ossification grades found in the collective in dependence of Brooker Score.

Frequencies of Brooker Score in dependency of Grade of Ossification							
variable	value	Grade 1		Grade 2		Grade 3	
		n	%*	n	%*	n	%*
Brooker Score	1	6	13.3	1	2.2		
	2	15	33.3	5	11.1		
	3	5	11.1	2	4.4		
	4	2	4.4	3	6.7	1	2.2
	Knee	5	11.1				

*The percentages are based on the number of non-missing values of both variables (n = 45).

).

**Table 3:** Potential risk factors for certain Grades of ossification.

Frequencies of categorical variables in dependency of Grade of Ossification						
variable	value	Grade 1 n = 33		Grade 2/3 n = 12		p value**
		n	%*	n	%*	
Gender	missing	6		2		
	male	12	75.0	4	25.0	1.000
	female	15	71.4	6	28.6	
Microbiology (operative)	no germs	26	74.3	9	25.7	1.000
	germs	7	70.0	3	30.0	
NSAR	missing			1		
	yes	23	82.1	5	17.9	0.169
	no	10	62.5	6	37.5	

*The percentages are based on the number of non-missing values of the row.

**p values of Fisher’s exact test.

**Table 4:** Influence of NSAID on Grade of Ossification, Necrosis and Osteomyelitis findings in tissue samples.

Frequencies of categorical variables in dependency of NSAID						
variable	Value	Yes (n = 32)		No (n = 18)		P value**
		n	%*	n	%*	
Grade of Ossification	missing	4		1		
	Grade 1	23	69.7	10	30.3	0.111
	Grade 2	5	45.5	6	54.5	
	Grade 3			1	100	
Necrosis	missing	4		1		
	No	28	65.1	15	34.9	0.137
	Yes			2	100	
Grade of Osteomyelitis	missing	4		1		
	II			1	100	0.137
	III			1	100	
	IV	28	65.1	15	34.9	

*The percentages are based on the number of non-missing values of the row.

**p values of Fisher’s exact test.

### Histological findings

We now present three exemplary cases of histological findings of intraoperatively taken tissue samples using the presented scoring system: **sample 1**: low-grade ossification, **sample 2**: high-grade ossification and **sample 3**: wear-induced synovitis, SLIM Type 1.

**Figure 3:**
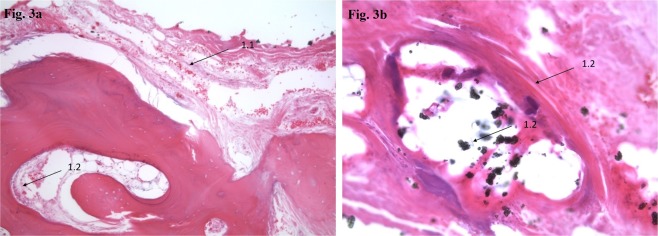
(**a & b**) H&E staining, original magnification approximately 200x. Sample 1: Ossification-Score: Grade 1 (Low bone formation, low bone destruction, low inflammatory infiltration), Necrosis (N-0: No Necrosis) and HOES (V: No signs of Osteomyelitis). Ossification Score formula: *A (1) B (0) C (V) D (PMMA and Zircondioxyde).*

Figure 3a & 3b (Sample 1): A/Ossification-Score (Grade 1: Low bone formation, low bone destruction, low inflammatory infiltration), B/Necrosis (no necrosis: 0) and  C/HOES (V: No signs of Osteomyelitis).

40 × 15 × 11 mm sized predominantly bony tissue with adherent fibrinous tissue. H&E staining, original magnification approximately 200x.

1.1 Bone-adherent fibrinous tissue with slightly increased cellularity.

**Figure 4:**
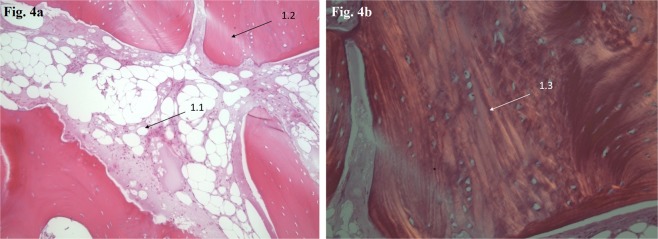
(**a &  b**) H&E staining, original magnification approximately 200x. Sample 2: Ossification-Score Grade 2: Moderate bone formation, moderate bone destruction, moderate inflammatory infiltration. Necrosis (N-1: Necrosis) and HOES (IV: Indicative for signs of chronic Osteomyelitis).  4a: Necrotic bone marrow with edema moderate inflammatory infiltration and osteoclasts.  4b: POL-analysis with irregular distribution of fibrils in a non-osteon like pattern as a prove for heterotopic ossification. Ossification Score formula: *A (2) B (1) Particle-identities (No) and C (IV).*
  15 × 12 × 5 mm sized tissue sample with minimal adherent fibrinous tissue. H&E staining, original magnification approximately 200x.
  1.1 Bone-adherent fibrinous tissue.

1.2 Fokal heterotopic ossification with intramedullary PMMA depositions (zirconium dioxide), few osteoclasts, low inflammatory infiltration with intramedullary edema and fibrosis.
  Figure 4a & 4b (Sample 2): A/Ossification-Score (Grade 2: Moderate bone formation, moderate bone destruction, moderate inflammatory infiltration) B/Necrosis (necrosis) und C/HOES (4 = (IV: Indicative for signs of chronic Osteomyelitis).

1.2 Irregulary contoured cartilage tissue.

1.3 Moderate bone formation with woven bone tissue (non-osteon bone) with edema, medullary fibrosis and moderate osteoblasts/intramedullary histiocyte infiltrates.

**Figure 5:**
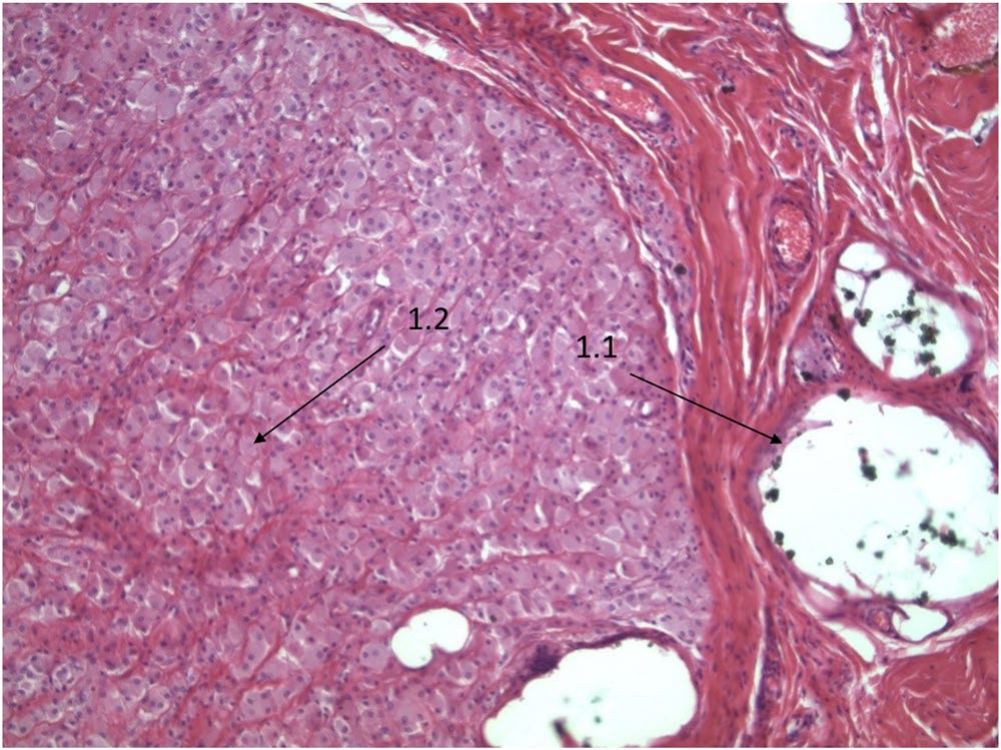
H&E staining, original magnification approximately 200x. Sample 3: SLIM-Type 1 wear particle type according to the SLIM-classification with microparticular PE-deposits in macrophages (left side) and PMMA as well as Zircondioxyde deposits in the partly depleted PMMA-vacuoles (right side). H&E staining, original magnification approximately 200x.

Figure 5 (Sample 3): SLIM-Type 1 wear particle type according to the consensus classification with microparticular PE-deposits in macrophages (left side) and PMMA as well as zirconium dioxide deposits in the partly depleted PMMA-vacuoles (right side).

11 × 16 × 3 mm size, predominantly fibrinous tissue showing initial indurations, no adherent osseous tissue. H&E staining, original magnification approximately 200x.

1.1 Collagenous, tense organized connective tissue with PMMA depositions (zirconium dioxide).

1.2 Periprosthetic Membrane of wear-induced type, SLIM-type 1.

## Discussion

We encountered 66% Grade I ossifications with neither necrosis nor osteomyelitis. These findings might be explained by a generally higher awareness for HO in orthopaedic and trauma surgery and therefore focused use of NSAIDs which are often prescribed not only for postoperative pain but also for an extended postoperative period for anti-inflammatory effects^31^. The majority of cases with ossification grades II & III were associated with septic revision cases (9/16 samples, 56.3%). This can be potentially explained by the fact that infections cause more significant inflammation and therefore are observed to have a higher incidence and grade of heterotopic ossifications. Manrique *et al*. have reported on more severe HO in septic revision cases^13^. The authors assumed that not only patient-related data such as an increased age, male gender or increased BMI may be causal for severe ossification, but that also surgery-related factors such as a higher tissue injury consisting of more aggressive and extensive soft tissue debridement, higher number of surgical procedures within short period of time (either due to treatment strategies with multiple procedures such as two-stage arthroplasty or because of persistent or recurrent PJI) and the lengthier surgical procedures lead to higher ossification grades. Rosteius *et al*. recently confirmed this suspicion and were able to demonstrate that chronic infections and multiple surgical interventions present significant risk factors for high-grade ossification^32^.

In the current study all tissue samples with findings of necrosis were associated with septic revision cases and positive intraoperative cultures. Though we could not demonstrate a correlation between positive intraoperative cultures and higher ossification grades, these two aspects of high-grade ossification on the one hand and necrosis findings on the other hand, suggest that extensive inflammation is caused by bacterial colonization and therefore more intense soft tissue reactions. It is also possible that a higher concentration of morphogenes might be triggered by a more radical surgical dissection and débridement in PJI cases resulting in greater surgical trauma and insult to the periarticular soft tissues.

Most tissue samples in this study were harvested from hip joints (44 samples, 88%), whereas 6 samples (12%) were taken from knee joints. Heterotopic ossification around the knee joint has already been observed before and presents a known long-term complication in orthopaedic knee surgery^26,33^. In the current study, the knee tissue samples were Grade I HO, even in the two septic revision cases. One potential explanation for these low-grade ossifications around the knee joint might be the lower soft tissue mass compared to the hip resulting in the release of a lower concentration of morphogenes which result in less ossification.

We also encountered 6 samples (12%) with radiological suspicion of HO which turned out to be SLIM-Type 1 wear-induced synovitis (WIS). Five of these samples were taken from hip joints, whereas one sample was taken from a knee joint. Since not only the vast majority of total hip and knee arthroplasties, but also all our inlays/onlays used in this study were made from polyethylene, wear is known to generate particles resulting in osteolysis which is a relatively common cause of revision surgery in total hip arthroplasty^34,35^. Polyethylene wear being a SLIM-Type I synovitis generating material has not been described so far and might have an influence on implant survivorship as well as on soft tissue reactions and ectopic bone formations.

Based on these results, we propose the first scoring system for Heterotopic Ossification according to intraoperatively taken tissue samples. This method might be a way to express histopathological findings in three simple letters and therethrough characterize the grade of ossification. Since this classification is applicable for all affected human joints suffering HO, it represents a further development of the Brooker classification^17^, which mainly relates to hip joints.

In conclusion, HO predominantly appears as low-grade ossifications in total hip and knee arthroplasty. Additionally, WIS is a special type of synovial infection that was associated with polyethylene inlays/onlays in all of the used implants (hip and knee protheses) in this study. A potential impact of this WIS on ossification gensis has to be clarified by further research.

The proposed scoring system might be helpful for grading HO according to intraoperative findings independent from the affected joint. Since it not only involves the grade of ossification but also captures necrotic tissue findings, this score is also applicable in more severe or longer ongoing infect situations. Lastly it also involves the HOES score for osteomyelitis^27^, which is a useful tool especially in septic revision cases. We know that sometime infections are not only limited to an implant infection including the periprosthetic membrane but also affect the adjacent, peri-implant bone which then leads to (chronic) osteomyelitis as it sometimes can be observed in long time low-grade infections^36–38^.

Limitations of this study are a relatively small sample size with just a small number of high-grade ossifications and missing records for potential preoperatively taken NSAID. Furthermore, we did not perform a postoperative follow-up to see if patients suffered HO development again and if it might be associated with an intraoperative ossification grade in any kind.

Further research is needed to investigate any potential correlation of HO activity and the grade of ossification. For activity measuring, scintigrams might help to graduate the metabolic activity of the chondral tissue. Lastly, clinical treatment suggestions should be worked out based on this activity level and HO grade.
